# Real-world predictors of relapse in patients with schizophrenia and schizoaffective disorder in a large health system

**DOI:** 10.1038/s41537-024-00448-2

**Published:** 2024-02-29

**Authors:** Anne Rivelli, Veronica Fitzpatrick, Michael Nelson, Kimberly Laubmeier, Courtney Zeni, Srikrishna Mylavarapu

**Affiliations:** 1 Advocate Aurora Research Institute, Milwaukee, IL USA; 2 Advocate Aurora Health, Milwaukee, IL USA; 3 Sumitomo Pharma America, Inc, Marlborough, MA USA

**Keywords:** Schizophrenia, Human behaviour

## Abstract

Schizophrenia is often characterized by recurring relapses, which are associated with a substantial clinical and economic burden. Early identification of individuals at the highest risk for relapse in real-world treatment settings could help improve outcomes and reduce healthcare costs. Prior work has identified a few consistent predictors of relapse in schizophrenia, however, studies to date have been limited to insurance claims data or small patient populations. Thus, this study used a large sample of health systems electronic health record (EHR) data to analyze relationships between patient-level factors and relapse and model a set of factors that can be used to identify the increased prevalence of relapse, a severe and preventable reality of schizophrenia. This retrospective, observational cohort study utilized EHR data extracted from the largest Midwestern U.S. non-profit healthcare system to identify predictors of relapse. The study included patients with a diagnosis of schizophrenia (ICD-10 F20) or schizoaffective disorder (ICD-10 F25) who were treated within the system between October 15, 2016, and December 31, 2021, and received care for at least 12 months. A relapse episode was defined as an emergency room or inpatient encounter with a pre-determined behavioral health-related ICD code. Patients’ baseline characteristics, comorbidities and healthcare utilization were described. Modified log-Poisson regression (i.e. log Poisson regression with a robust variance estimation) analyses were utilized to estimate the prevalence of relapse across patient characteristics, comorbidities and healthcare utilization and to ultimately identify an adjusted model predicting relapse. Among the 8119 unique patients included in the study, 2478 (30.52%) experienced relapse and 5641 (69.48%) experienced no relapse. Patients were primarily male (54.72%), White Non-Hispanic or Latino (54.23%), with Medicare insurance (51.40%), and had baseline diagnoses of substance use (19.24%), overweight/obesity/weight gain (13.06%), extrapyramidal symptoms (48.00%), lipid metabolism disorder (30.66%), hypertension (26.85%), and diabetes (19.08%). Many differences in patient characteristics, baseline comorbidities, and utilization were revealed between patients who relapsed and patients who did not relapse. Through model building, the final adjusted model with all significant predictors of relapse included the following variables: insurance, age, race/ethnicity, substance use diagnosis, extrapyramidal symptoms, number of emergency room encounters, behavioral health inpatient encounters, prior relapses episodes, and long-acting injectable prescriptions written. Prevention of relapse is a priority in schizophrenia care. Challenges related to historical health record data have limited the knowledge of real-world predictors of relapse. This study offers a set of variables that could conceivably be used to construct algorithms or models to proactively monitor demographic, comorbidity, medication, and healthcare utilization parameters which place patients at risk for relapse and to modify approaches to care to avoid future relapse.

## Background

Schizophrenia and schizoaffective disorder are two severe, chronic psychiatric disorders characterized by major impairments in a person’s ability to perceive, feel, and act^1^. While they remain separable in diagnostic systems, they are largely indistinguishable on key measures; thus, much of what we know about schizophrenia reflects both disorders^2^. The most recent systematic review of schizophrenia reveals a prevalence of about 1%^3–5^. Despite its relative rarity, schizophrenia is one of the top 15 leading causes of disability worldwide^6^ and one of the most expensive mental disorders to treat^7–9^. Costs associated with treatment received as a result of relapse account for the largest share of treatment costs in schizophrenia, making prevention of relapse a top priority in care^10–13^.

Schizophrenia is often characterized by recurring relapses^9,14^, with up to 81.9% of individuals with schizophrenia or schizoaffective disorder experiencing relapse within five years of their diagnosis^15^. Rates of subsequent relapse are similarly high^15^. A relapse in patients with schizophrenia can represent a variety of health escalations, including an acute exacerbation of schizophrenia symptoms leading to cascading events like medication non-adherence, major life disruptions such as a job or legal issues, self-harm and hospitalization^16,17^. In the U.S., it has been estimated that $2 billion is spent annually for hospitalizations associated with relapse of patients with schizophrenia^10^. To mitigate this, intensive and costly interventions have been specifically designed to help prevent or minimize relapse^10^. However, given the cost of these interventions and the progressive nature of schizophrenia relapse^18^, it is critical to identify those most at risk of relapse.

Medication non-adherence is most consistently documented as a predictor of relapse^12,15,16,19,20^, particularly a first relapse^21^. While at least one relapse is typical among individuals with diagnosed schizophrenia, prior relapse is perhaps the strongest predictor of subsequent relapse^9^. Additional predictors of relapse include medical and psychiatric comorbid conditions, like diabetes^22^, medication side effects and substance use^14,20^. Comorbid conditions can lead to relapse due to medication non-adherence or by way of symptom exacerbation. There is also evidence that life stressors, quality of life, functioning and insight level are associated with relapse in schizophrenia^9^, but these findings are mixed due to difficulties in elucidating relationships between these factors and the more tangible predictors of relapse^14^.

Aside from the immense mental and emotional effects of relapse in the personal lives of individuals with schizophrenia and schizoaffective disorders, there are significant logistical and financial consequences, specifically, increased healthcare utilization and associated medical costs^23–25^. Due to the chronic and severe nature of the disease, people with schizophrenia tend to be high utilizers of the healthcare system in general. The economic burden of schizophrenia in the U.S. in 2019 was estimated at $343.2 billion, which corresponds to an annual excess cost per person with schizophrenia of $87,856^26^. Schizophrenia-related relapse is a major factor in generating both high hospitalization rates and associated costs. Patient costs after recent relapse were found to be 3-4 times the costs for patients without recent relapse, with inpatient costs being 5 times higher among those with recent relapse^9^. This is supported by another study revealing an average of 57.8 days of inpatient care utilized by those who recently relapsed, compared to 0 days among those who did not recently relapse^13^. Increased healthcare resource utilization, including duration of hospital stay, number of encounters with providers, and total costs, have been documented up to one year after an episode of relapse^23^. Given evidence that medication non-adherence is associated with relapse, it is no surprise that individuals with schizophrenia who fail to adhere to their medication regime are 1.5 times more likely to utilize healthcare inpatient services^19^.

Despite existing knowledge of common predictors of relapse in schizophrenia, most prior studies rely on insurance claims data and/or small sample patient populations, and thus there remains a need to look at larger, more diverse populations from a health systems perspective to add to this important body of research. The objective of this study was to analyze the relationship between patient-level factors and relapse, using electronic health record (EHR) data to better inform both clinicians and the research community of ways to predict and ultimately prevent relapse. EHR data has unique advantages over other data sources, specifically that is provides a more real-time view of patient’s health and care, offers more clinical depth from patient care settings and includes populations from various insurance types as well as those who are uninsured. While definitions of relapse vary in the literature, a systematic review of definitions of relapse in schizophrenia concluded that hospitalization was the most common feature of proxy definitions used to identify schizophrenia relapse^27^; thus, this study defined relapse similarly, which is further described below.

## Methods

**Fig. 1:**
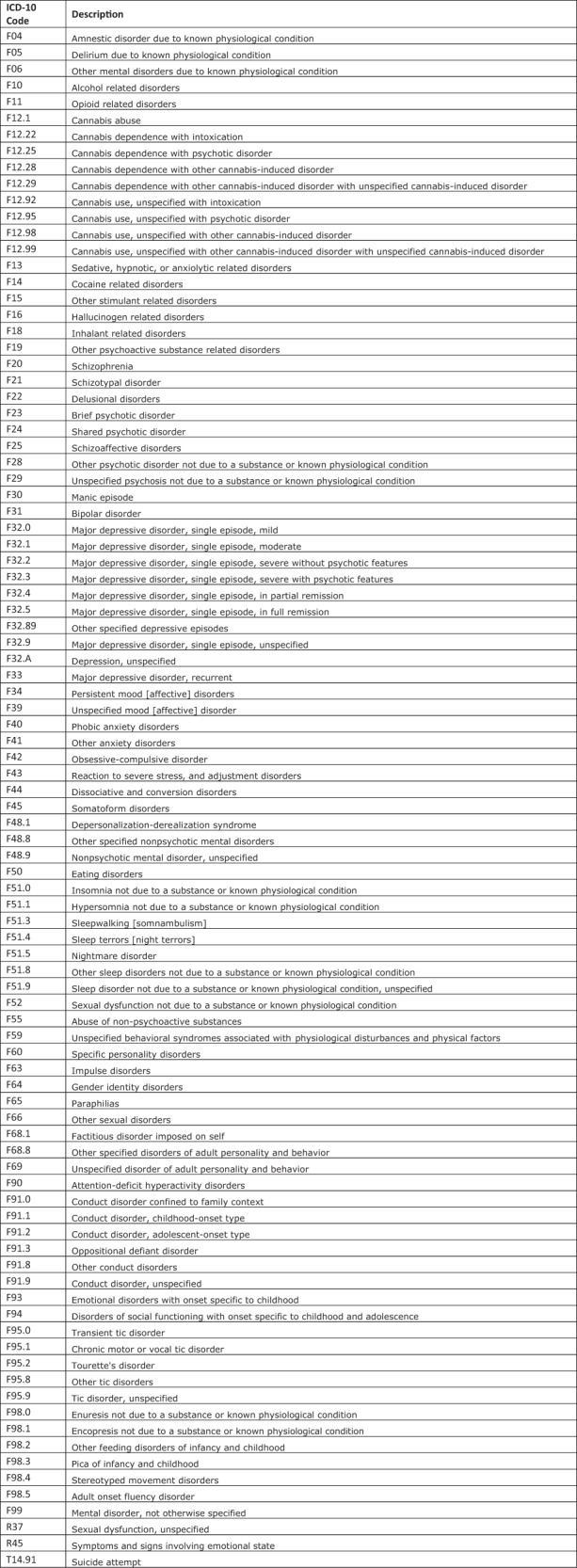
ICD-10 Codes Used to Define and Identify Behavioral Health-Related Psychiatric Services Indicating Relapse.

This retrospective, observational cohort study utilized EHR data extracted from the largest Midwestern non-profit healthcare system to explore healthcare utilization and outcomes among patients with schizophrenia and schizoaffective disorder. The study included patients with an International Classification of Diseases, Tenth Revision (ICD-10) diagnosis code^28^ for schizophrenia (F20) and/or schizoaffective disorder (F25) within the healthcare system’s EHR between October 15, 2016 and December 31, 2021 and at least one additional encounter at least 12 months later. The Index Date was defined as the patient’s first encounter date with a diagnosis of schizophrenia and/or schizoaffective disorder within the designated timeframe. Data across all available patient encounters in the system up to three years after their Index Date or December 31, 2021, whichever was first, were extracted. This sample was chosen a priori in efforts to adequately assess potential predictors throughout a 6-month baseline period (Index Date to 6 months post-Index Date) and to understand relapse prevalence throughout a 6+ month post-baseline period (6 months to 1+ year(s) post-Index Date). The outcome of interest in this study was relapse, as represented by the first relapse after the 6-month baseline period within the system. In this study, relapse was defined as any inpatient or emergency room encounter (i.e. hospitalization) occurring after the Index Date with an associated behavioral health (BH) ICD-10 code as listed in Fig. 1. BH ICD-10 codes were adapted from published literature reflecting a comprehensive list of psychiatric-related healthcare services^29^. This study was reviewed and approved by the healthcare system’s Institutional Review Board (#22.092ET).

### Data

**Fig. 2:**
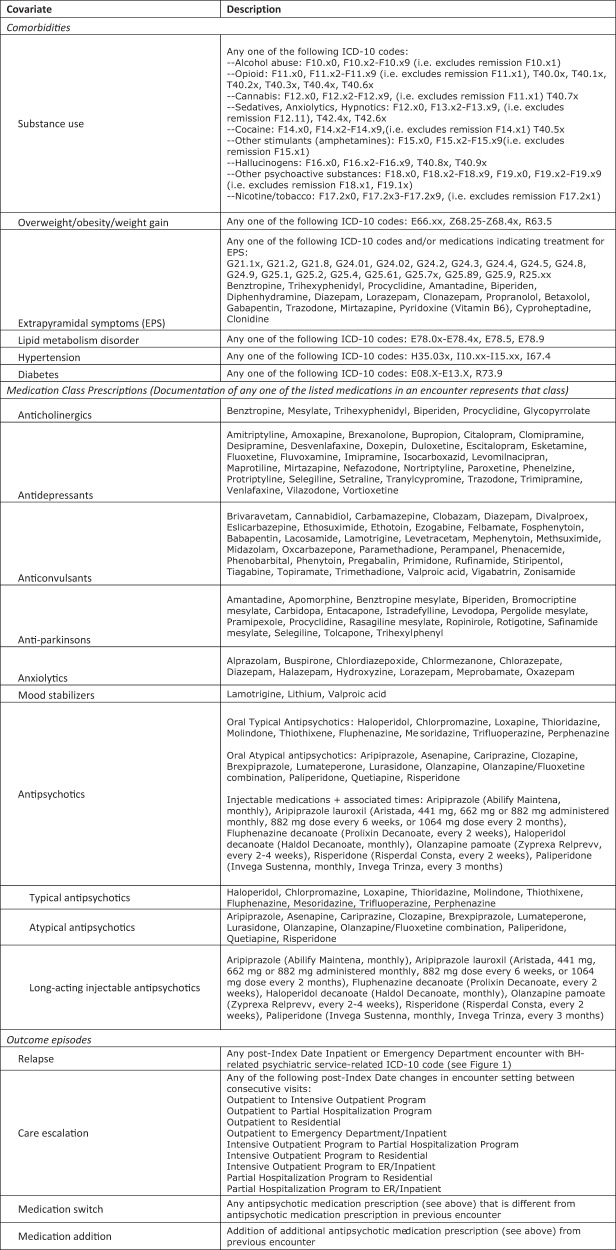
Descriptions of All Candidate Covariates Measured as Documented (Yes/No) in Any Encounter(s) between Patients’ Index Date and 6 Months Post-Index Date.

Demographic variables at Index Date were collected on all sample patients, including: Age (continuous); Race/ethnicity (categorical), White Non-Hispanic or Latino (NH), Black NH, Asian NH, American Indian or Alaskan Native NH, Other Pacific Islander NH, Two or more races NH, and Hispanic or Latino; Insurance type (categorical), private, Medicare, Medicaid, other; Sex (categorical), male or female; Index Date diagnosis (categorical), schizophrenia, schizoaffective, both; and Index Date setting of care (categorical), outpatient, inpatient, emergency room, BH (inpatient or other). Additionally, variables indicating healthcare utilization, treatment and outcomes were collected across all encounters between their Index Date and 6 months post-Index Date on sample patients, including Comorbidities (yes/no), substance use, overweight/obesity/weight gain, extrapyramidal symptoms (EPS), lipid metabolism disorder, hypertension, diabetes; medication class prescriptions (yes/no), anticholinergic, antidepressant, anticonvulsant, anti-Parkinson, anxiolytic, mood stabilizer, antipsychotics (typical, atypical, long-acting injectable (LAI)); encounter setting of care (yes/no), outpatient, inpatient, emergency room, BH (inpatient or other); and episodes of outcomes (yes/no), relapse, care escalation, medication switch, medication addition. The number of medication class prescriptions, encounter settings of care, and outcome episodes were summed for each unique patient between their Index Date and six months post-Index Date and used as continuous variables in modeling. It should be noted that, despite great efforts, medication non-adherence was unable to be extracted for analysis in this study. Finally, while Index Date diagnosis and Index Date setting of care were described in bivariate analysis, they were not used in modeling. Neither accuracy nor consistency of Index Date diagnosis could be verified in this prevalence dataset. Further, since this was a prevalence dataset, the Index Date setting of care was deemed uninterpretable given this encounter could represent an arbitrary timepoint in their disease and care. See Fig. 2 for in-depth definitions of each data variable.

### Statistical methods

Data management and analysis of the sample were conducted with SAS statistical software (Version 9.4; SAS Institute, Cary, NC). All analyses of the sample data were performed by research personnel employed by the health system. Categorical patient demographics at Index Date and comorbidities diagnosed within the first 6 months of follow-up within the system were described as counts (percentages), overall and by outcome (i.e. relapse) status. Modified log-Poisson regression analyses were performed to generate prevalence ratios (PR) with corresponding 95% confidence intervals (CI) reflecting the relative prevalence of patients experiencing relapse after 6 months of follow-up in the system as compared to the reference group of patients (those not experiencing relapse after 6 months of follow-up in the system). P-values reflect statistically significant differences in prevalence ratios between participant groups. Continuous variables, including follow-up time in months, patient age, numbers of healthcare utilization encounters, outcome episodes and medication class prescriptions, were described as means with standard deviations and medians with interquartile ranges. Student’s t-tests were performed to generate mean differences but, given all were skewed, Mann-Whitney U (or Wilcoxon Rank Sum) tests were performed to generate p-values reflecting statistically significant differences in medians between participant groups.

Given the outcome in this study was not rare (30.52% prevalence), modified log-Poisson regression analysis (i.e. log Poisson regression with a robust variance estimation) was utilized to model the prevalence of relapse as a binary outcome. It is well-documented that risk ratio is preferred over odds ratio in appropriate situations^30–33^; further, research supports the use of this modified regression analysis approach to obtain consistent and reliable risk ratio estimates and corresponding confidence^30,32^. In this study utilizing prevalence data, risk ratios represent prevalence ratios and will be referred to as such. Finally, to generate robust error variances, generalized estimating equations were utilized; thus, Quasi-likelihood information criterion (QIC) goodness of fit statistics were assessed for the goodness of fit of models in model building.

To identify important covariates predicting prevalence of relapse, all variables, except inclusion diagnosis and setting of care, were considered candidate covariates to include. First, collinearity, as defined by *r* > 0.70, between all variables was assessed. The remaining candidate covariates were added to the model one-by-one. A variable remained in the model for a subsequent step if it, or any level of it if categorical, was statistically significant at *p* < 0.05. At each step, parameter estimates were reviewed and all variables in the model were re-assessed for statistical significance at *p* < 0.05. Specifically, if a previously significant variable became statistically insignificant with the addition of a new statistically significant variable, the insignificant variable was removed from the model at that step. Adjusted models were compared to the complete adjusted model with all candidate covariates and a final adjusted model that best reflects the most parsimonious group of significant predictors of relapse was identified.

## Results

To explore the outcome of interest (relapse) among the study sample, patients were grouped as those who experienced at least one relapse after the 6-month baseline period (“relapse”) or those who experienced zero relapses after the 6-month baseline period (“no relapse”). Among the 8119 unique patients with at least 12 months of data, 2478 (30.52%) experienced relapse and 5641 (69.48%) experienced no relapse. Among the overall sample included in this study, at Index Date, patients were primarily male (54.72%), White Non-Hispanic or Latino (54.23%), with Medicare insurance (51.40%), a diagnosis of schizophrenia (53.42%), and mostly presented to an outpatient setting at Index Date (52.63%). In the 6-month baseline period, patients received diagnoses of substance use (19.24%), overweight/obesity/weight gain (13.06%), EPS (48.00%), lipid metabolism disorder (30.66%), hypertension (26.85%), and diabetes (19.08%).

Differences by relapse status revealed that, relative to those who did not experience relapse after the baseline period, patients who experienced relapse had, on average, 1.22[0.87,1.57] more months of follow-up (*p* < 0.0001) and were *more likely* to: be Black Non-Hispanic or Latin (NH) (PR = 1.26[1.17, 1.35], *p* < 0.0001), Other Pacific Islander NH (PR = 2.12(1.01, 4.46], *p* = 0.0474), Two or more races NH (PR = 1.43[1.07, 1.93], *p* = 0.0175), and Hispanic or Latino (PR = 1.29[1.12, 1.48], *p* = 0.0005) compared to White NH; have Medicare insurance (PR = 1.16[1.02,1.32], *p* = 0.0256) and Medicaid insurance (PR = 1.68[1.47, 1.91], *p* < 0.0001) compared to private insurance; have a diagnosis of schizoaffective disorder (PR = 1.14[1.07, 1.22], *p* < 0.0001) compared to schizophrenia; have diagnoses of substance use (PR = 1.74[1.62,1.86], *p* < 0.0001) and EPS (PR = 2.20[2.05, 2.36], *p* < 0.0001). Patients who experienced relapse were *less likely* to have diagnoses of: overweight/obesity/weight gain (PR = 0.81[0.73,0.91], *p* = 0.0002), lipid metabolism disorder (PR = 0.80[0.74,0.87], *p* < 0.0001), hypertension (PR = 0.83[0.77,0.90], *p* < 0.0001), and diabetes (PR = 0.88[0.81, 0.96], *p* = 0.0058). There was no difference in sex by relapse status.

Within the 6-month baseline period, patients who experienced relapse generally had more healthcare encounters, episodes of outcomes of interest, and medication class prescriptions. Specifically, patients who experienced relapse had 0.11 more inpatient (95%CI:[0.08, 0.14], *p* < 0.0001), 1.68 more emergency room (95%CI:[1.48,1.88], *p* < 0.0001), and 1.15 more BH (95%CI:[0.89, 1.41], *p* < 0.0001) encounters, including 0.39 BH inpatient (95%CI:[0.35, 0.43], *p* < 0.001) encounters. There were no differences in outpatient or BH outpatient encounters by relapse status. On average, patients who experienced relapse experienced 1.14 more relapse episodes during the baseline period (95%CI:[1.03, 1.25], *p* < 0.0001), 1.58 more care escalations (95%CI:[1.37, 1.78], *p* < 0.0001), 0.39 more medication switches (95%CI:[0.34, 0.43], *p* < 0.0001), and 0.34 more medication additions (95%CI:[0.30, 0.38], *p* < 0.0001). Finally, on average, patients who experienced relapse were written more prescriptions across all medication classes, specifically 1.48 more anticholinergics (95%CI:[1.20, 1.75], *p* < 0.0001), 2.65 more antidepressants (95%CI:[2.24,3.06], *p* < 0.0001), 2.81 more anticonvulsants (95%CI:[2.28, 3.33], *p* < 0.0001), 1.46 more anti-parkinson prescriptions (95%CI:[1.20, 1.72], *p* < 0.0001), 2.48 more anxiolytics (95%CI:[2.11, 2.84], *p* < 0.0001), 0.75 more mood stabilizers (95%CI:[0.53, 0.97], *p* < 0.0001), 1.26 more typical antipsychotics (95%CI:[1.04,1.48], *p* < 0.0001), 3.63 more atypical antipsychotics (95%CI:[3.23,4.03], *p* < 0.0001), and 0.13 more LAI antipsychotics (95%CI:[0.03, 0.22), *p* = 0.0006). See Table 1 for complete patient demographics and differences by relapse status.

**Table 1:** Demographics of Sample at Index Date and Utilization and Outcomes from Index Date to 6 Months of F/U, by Outcome (Relapse) Status.

Demographic Variables	Overall (*N* = 8119)	Relapse		Prevalence risk^#^	*P* value
		Relapse (*N* = 2478; 30.52%)	No Relapse (*N* = 5641; 69.48%)		
*Inclusion Diagnosis*					
Schizophrenia	4337 (53.42%)	1233 (28.43%)	3104 (71.57%)	REF	–
Schizoaffective	3712 (45.72%)	1206 (32.49%)	2506 (67.51%)	1.14 (1.07, 1.22)	<0.0001
Both^	70 (0.86%)	39 (55.71%)	31 (44.29%)		
*Insurance Type*					
Private	889 (10.95%)	206 (23.17%)	683 (76.83%)	REF	–
Medicare	4173 (51.40%)	1121 (26.86%)	3052 (73.14%)	1.16 (1.02, 1.32)	0.0256
Medicaid	2761 (34.01%)	1072 (38.83%)	1689 (61.17%)	1.68 (1.47, 1.91)	<0.0001
Other	296 (3.65%)	79 (26.69%)	217 (73.31%)	1.15 (0.92, 1.44)	0.2154
*Sex*					
Female	3676 (45.28%)	1114 (30.30%)	2562 (69.70%)	REF	–
Male	4443 (54.72%)	1364 (30.70%)	3079 (69.30%)	1.01 (0.94, 1.10)	0.7483
*Race/Ethnicity (N* = *8082)*					
White, Non-Hispanic or Latino	4383 (54.23%)	1206 (27.52%)	3177 (72.48%)	REF	-
Black, Non-Hispanic or Latino	2653 (32.83%)	920 (34.68%)	1733 (65.32%)	1.26 (1.17, 1.35)	<0.0001
Asian, Non-Hispanic or Latino	166 (2.05%)	44 (26.51%)	122 (73.49%)	0.96 (0.74, 1.30)	0.8076
American Indian or Alaskan Native, Non-Hispanic or Latino	95 (1.18%)	23 (24.21%)	72 (75.79%)	0.88 (0.58, 1.33)	0.5433
Other Pacific Islander, Non-Hispanic or Latino	12 (0.15%)	7 (58.33%)	5 (41.67%)	2.12 (1.01, 4.46)	0.0474
Two or more races, Non-Hispanic or Latino	114 (1.41%)	45 (39.47%)	69 (60.53%)	1.43 (1.07, 1.93)	0.0175
Hispanic or Latino	659 (8.15%)	233 (35.36%)	426 (64.64%)	1.29 (1.12, 1.48)	0.0005
*Index Date Setting of Care*					
Outpatient	4273 (52.63%)	968 (22.65%)	3305 (77.35%)	REF	–
Inpatient	188 (2.32%)	104 (55.32%)	84 (44.68%)	2.44 (2.12, 2.81)	<0.0001
Emergency Room	855 (10.53%)	470 (54.97%)	385 (45.03%)	2.43 (2.23, 2.63)	<0.0001
Behavioral Health	2803 (34.52%)	936 (33.39%)	1867 (66.61%)	1.47 (1.37, 1.59)	<0.0001
Behavioral Health Other	2272 (81.06%)	561 (24.69%)	1711 (75.31%)	REF	REF
Behavioral Health Inpatient	531 (18.94%)	375 (70.62%)	156 (29.38%)	2.86 (2.61, 3.13)	<0.0001
*Age (Mean (SD); Median (IQR))*^*#*^	47.45 (15.66); 49 (35–59)	44.67 (15.35); 45 (32–56)	48.67 (15.64); 51 (36–60)	−4.00 (−4.72, −3.27)	<0.0001
*Categories*					
0–17	99 (1.22%)	28 (28.28%)	71 (71.72%)	REF	–
18–64	6895 (84.92%)	2201 (31.92%)	4694 (68.08%)	1.44 (1.29, 1.62)	<0.0001
65+	1125 (13.86%)	249 (22.13%)	876 (77.87%)	1.13 (0.82, 1.55)	0.4522
*Comorbidities*					
Substance Use	1562 (19.24%)	725 (46.41%)	837 (53.59%)	1.74 (1.62, 1.86)	<0.0001
Overweight/Obesity/Weight Gain	1060 (13.06%)	270 (25.47%)	790 (74.53%)	0.81 (0.73, 0.91)	0.0002
EPS	3897 (48.00%)	1660 (42.60%)	2237 (57.40%)	2.20 (2.05, 2.36)	<0.0001
Disorders of lipid metabolism	2489 (30.66%)	649 (26.07%)	1840 (73.93%)	0.80 (0.74, 0.87)	<0.0001
Hypertension	2180 (26.85%)	578 (26.51%)	1602 (73.49%)	0.83 (0.77, 0.90)	<0.0001
Diabetes	1549 (19.08%)	427 (27.57%)	1122 (72.43%)	0.88 (0.81, 0.96)	0.0058

Healthcare Utilization and Outcomes (Mean (SD); Median (IQR))	Overall (*N* = 8119)	Relapse		Mean Difference +	*P* value
		Relapse (*N* = 2478; 30.52%)	No relapse (*N* = 5641; 69.48%)		
*Total Follow-up (Months)*	27.99 (7.60); 31 (22–35)	28.84 (7.38); 32 (23–35)	27.62 (7.67); 30 (22–35)	1.22 (0.87, 1.57)	<0.0001
*Utilization as Encounters*					
Outpatient	6.81 (10.30); 4 (1–8)	7.81 (12.41); 4 (1–9)	6.38 (9.19); 4 (1–8)	1.43 (0.89, 1.97)	0.1876
Inpatient	0.16 (0.56); 0 (0–0)	0.24 (0.69); 0 (0–0)	0.13 (0.49); 0 (0–0)	0.11 (0.08, 0.14)	<0.0001
Emergency Room	0.97 (3.22); 0 (0–1)	2.13 (4.98); 1 (0–2)	0.45 (1.78); 0 (0–0)	1.68 (1.48, 1.88)	<0.0001
Behavioral Health	2.00 (4.61); 0 (0–2)	2.80 (6.12); 1 (0–3)	1.65 (3.70); 0 (0–2)	1.15 (0.89, 1.41)	<0.0001
Behavioral Health Inpatient	0.18 (0.65); 0 (0–0)	0.46 (1.02); 0 (0–1)	0.06 (0.33); 0 (0–0)	0.39 (0.35, 0.43)	<0.0001
Behavioral Health Other	1.82 (4.48); 0 (0-2)	2.35 (5.91); 0 (0–2)	1.59 (3.65); 0 (0–2)	0.76 (0.51, 1.01)	0.4917
*Outcomes as Episodes*					
Prior Relapse Episodes	0.52 (1.71); 0 (0–0)	1.31 (2.67); 0 (0–1)	0.17 (0.83); 0 (0–0)	1.14 (1.03, 1.25)	<0.0001
Care Escalation Episodes	1.40 (3.51); 0 (0–1)	2.50 (4.91); 1 (0–3)	0.92 (2.52); 0 (0–1)	1.58 (1.37, 1.78)	<0.0001
Medication Switch Episodes	0.24 (0.71); 0 (0–0)	0.51 (1.03); 0 (0–1)	0.12 (0.46); 0 (0–0)	0.39 (0.34, 0.43)	<0.0001
Medication Addition Episodes	0.21 (0.65); 0 (0–0)	0.44 (0.97); 0 (0–1)	0.10 (0.41); 0 (0–0)	0.34 (0.30, 0.38)	<0.0001
*Medication Categories*					
Anticholinergics	1.24 (4.87); 0 (0–0)	2.27 (6.47); 0 (0–2)	0.79 (3.88); 0 (0–0)	1.48 (1.20, 1.75)	<0.0001
Antidepressants	2.95 (7.49); 0 (0–2)	4.80 (9.69); 0 (0–5)	2.15 (6.12); 0 (0–1)	2.65 (2.24, 3.06)	<0.0001
Anticonvulsants	3.55 (9.53); 0 (0–2)	5.50 (12.12); 0 (0–5)	2.70 (7.98); 0 (0–1)	2.81 (2.28, 3.33)	<0.0001
Anti-parkinson	1.19 (4.55); 0 (0–0)	2.21 (6.22); 0 (0-2)	0.75 (3.48); 0 (0–0)	1.46 (1.20, 1.72)	<0.0001
Anxiolytics	2.31 (6.49); 0 (0–2)	4.03 (8.54); 1 (0–4)	1.55 (5.17); 0 (0–0)	2.48 (2.11, 2.84)	<0.0001
Mood stabilizers	0.68 (3.70); 0 (0–0)	1.21 (5.38); 0 (0–0)	0.45 (2.61); 0 (0–0)	0.75 (0.53, 0.97)	<0.0001
Typical antipsychotics	1.04 (3.81); 0 (0–0)	1.92 (5.32); 0 (0–2)	0.66 (2.82); 0 (0–0)	1.26 (1.04, 1.48)	<0.0001
Atypical antipsychotics	4.24 (8.65); 1 (0–5)	6.77 (11.41); 3 (0–8)	3.13 (6.82); 0 (0–4)	3.63 (3.23, 4.03)	<0.0001
LAI antipsychotics	0.14 (1.51); 0 (0–0)	0.23 (2.27); 0 (0–0)	0.10 (1.01); 0 (0–0)	0.13 (0.03, 0.22)	0.0006

^#^Prevalence Risk (PR) and *P* value for categorical variables generated from modified log-Poisson with sandwich estimation.

+Mean difference for continuous variables generated from Student’s *t*-test; *P* value for continuous variables generated from Mann-Whitney U test due to skewness (except age and total follow-up).

Before model building, Pearson correlation analyses showed BH encounters were correlated with BH outpatient encounters (*r* = 0.99), medication addition episodes were correlated with medication switch episodes (*r* = 0.81) and anti-parkinson prescriptions were correlated with anticholinergic prescriptions (*r* = 0.74); subsequently, after consultation with the research team, BH encounters, medication addition episodes and anti-parkinson prescriptions were not included as candidate covariates. After the model-building process was complete, the final adjusted model of significant predictors of relapse included the following variables: insurance, age, race/ethnicity, substance use diagnosis, EPS diagnosis and, during the 6-month baseline period prior to relapse, the number of emergency room encounters, BH inpatient encounters, prior relapses episodes, LAI prescriptions written. When adjusting for the remaining covariates in this final adjusted model, these are the specific findings regarding relapse prevalence:

- Insurance: Relative to those with private insurance, patients with Medicaid and Medicare insurances were at 33% and 22% increased prevalence, respectively (PR = 1.33[1.17,1.51], *p* < 0.001 and PR = 1.22[1.08,1.39], *p* = 0.0018, respectively).
- Age: With each one-year increase, there was a 1% reduction in prevalence (PR = 0.99[0.99,0.99], *p* < 0.0001).
- Race/ethnicity: Relative to those who are White NH, patients who were Hispanic or Latino had 15% increased prevalence (PR = 1.15[1.03,1.28], *p* = 0.0121) and patients who are Other Pacific Islander NH had an 83% increased prevalence (PR = 1.83[1.16,2.89], *p* = 0.0090); also, patients who were American Indian or Alaskan Native NH had a 26% reduction in relapse prevalence relative to those who were White NH (PR = 0.74[0.54,1.00], *p* = 0.0512).
- Substance use diagnosis: Relative to those without a diagnosis for substance use, patients with a diagnosis of substance use had 33% increased prevalence (PR = 1.33[1.24,1.43], *p* < 0.0001).
- EPS diagnosis: Relative to those without a diagnosis code representing EPS, patients with an EPS diagnosis had a 78% increase in prevalence (PR = 1.78[1.65,1.92], *p* < 0.0001).
- Emergency room encounters: With each one-unit increase in encounters, there was a 2% increase in prevalence (PR = 1.02[1.01,1.02], *p* < 0.0001).
- BH inpatient encounters: With each one unit increase in encounters, there was a 10% increase in prevalence (PR = 1.10[1.06,1.14], *p* < 0.0001).
- Prior relapses: With each one unit increase in prior relapse experienced, there was a 5% increase in prevalence (PR = 1.05[1.03,1.06], *p* < 0.0001).
- LAI prescriptions: With each LAI prescription written, there was a 1% increase in prevalence (PR = 1.01[1.00,1.02], *p* = 0.0349).

**Fig. 3:**
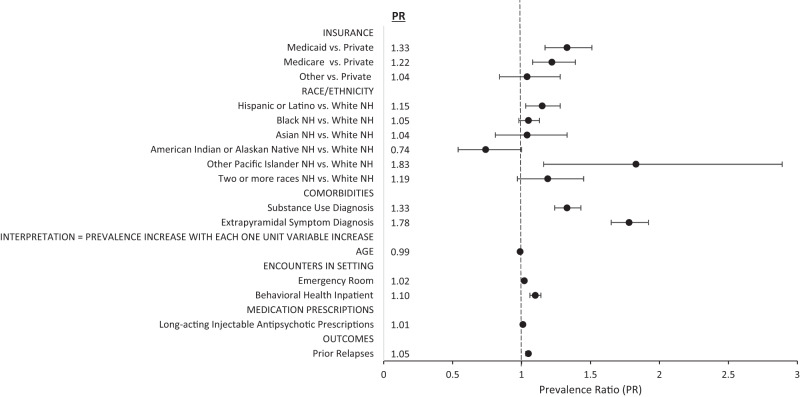
Final Abjusted Model Representing Signficant Predictors of Relapse Among AAH Patients with Schizophrenia or Schizoaffective Disorder.

Crude and adjusted model parameters can be viewed in Table 2 and, visually, in Fig. 3.

**Table 2:** Crude and Adjusted Parameter Estimates of Candidate and Final Adjusted Model Covariates, Respectively.

	Crude Models			Final Adjusted Model		
Model Parameters	β (SE)	Prevalence Ratio (PR)	P-value	β (SE)	Prevalence Ratio (PR)	*P*-value
QIC^^^				15022.4075		
Intercept	–	–	–	−1.5645 (0.0784)	–	<0.0001
**Candidate Covariates**^**+**^						
*Insurance*						
Private	–	REF	–	–	REF	–
Medicaid	0.5162 (0.0656)	1.68 (1.47, 1.91)	<0.0001	0.2859 (0.0640)	1.33 (1.17, 1.51)	<0.0001
Medicare	0.1478 (0.0662)	1.16 (1.02, 1.32)	0.0256	0.2026 (0.0649)	1.22 (1.08, 1.39)	0.0018
Other	0.1413 (0.1141)	1.15 (0.92, 1.44)	0.2154	0.0369 (0.1075)	1.04 (0.84, 1.28)	0.7311
* Age*	-0.0113 (0.0013)	0.99 (0.99, 0.99)	<0.0001	-0.0075 (0.0012)	0.99 (0.99, 0.99)	<0.0001
*Race/Ethnicity*						
White, NH	–	REF	–	–	REF	–
American Indian or Alaskan Native, NH	-0.1280 (0.2105)	0.88 (0.58, 1.33)	0.5433	-0.3043 (0.1560)	0.74 (0.54, 1.00)	0.0512
Asian, NH	-0.0374 (0.1535)	0.96 (0.74, 1.30)	0.8076	0.0377 (0.1272)	1.04 (0.81, 1.33)	0.7667
Black, NH	0.2314 (0.0438)	1.26 (1.16, 1.357	<0.0001	0.0481 (0.0362)	1.05 (0.98, 1.13)	0.1837
Hispanic or Latino	0.2507 (0.0716)	1.29 (1.12, 1.48)	0.0005	0.1376 (0.0549)	1.15 (1.03, 1.28)	0.0121
Other Pacific Islander, NH	0.7514 (0.3791)	2.12 (1.01, 4.46)	0.0474	0.6067 (0.2322)	1.83 (1.16, 2.89)	0.0090
Two or more races, NH	0.3609 (0.1518)	1.43 (1.07, 1.93)	0.0175	0.1705 (0.1015)	1.19 (0.97, 1.45)	0.0931
*Emergency Room Encounters*	0.0381 (0.0022)	1.04 (1.03, 1.04)	<0.0001	0.0161 (0.0032)	1.02 (1.01, 1.02)	<0.0001
*Behavioral Health Inpatient Encounters*	0.2802 (0.0137)	1.32 (1.29, 1.36)	<0.0001	0.0928 (0.0181)	1.10 (1.06, 1.14)	<0.0001
*Prior Relapses Episodes*	0.1194 (0.0054)	1.13 (1.11, 1.14)	<0.0001	0.0460 (0.0078)	1.05 (1.03, 1.06)	<0.0001
*LAI Prescriptions*	0.0228 (0.0080)	1.02 (1.01, 1.04)	0.0045	0.0100 (0.0047)	1.01 (1.00, 1.02)	0.0349
*Substance Use Diagnosis*	0.5517 (0.0442)	1.74 (1.59, 1.89)	<0.0001	0.2866 (0.0353)	1.33 (1.24, 1.43)	<0.0001
*Extrapyramidal Symptoms*	0.7878 (0.0427)	2.20 (2.02, 2.39)	<0.0001	0.5746 (0.0385)	1.78 (1.65, 1.92)	<0.0001
*Outpatient Encounters*	0.0077 (0.0016)	1.01 (1.00, 1.01)	<0.0001	–	–	–
*Inpatient Encounters*	0.1768 (0.0260)	1.19 (1.13, 1.26)	<0.0001	–	–	–
*Behavioral Health Other Encounters*	0.0200 (0.0034)	1.02 (1.01, 1.03)	<0.0001	–	–	–
*Care Escalation Episodes*	0.0462 (0.0030)	1.05 (1.04, 1.05)	<0.0001	–	–	–
*Medication Switch Episodes*	0.3016 (0.0162)	1.35 (1.31, 1.40)	<0.0001	–	–	–
*Anticholinergic Prescriptions*	0.0222 (0.0022)	1.02 (1.02, 1.03)	<0.0001	–	–	–
*Antidepressant Prescriptions*	0.0208 (0.0017)	1.02 (1.02, 1.02)	<0.0001	–	–	–
*Anticonvulsant Prescriptions*	0.0137 (0.0014)	1.01 (1.01, 1.02)	<0.0001	–	–	–
*Anxiolytic Prescriptions*	0.0222 (0.0017)	1.02 (1.02, 1.03)	<0.0001	–	–	–
*Mood Stabilizer Prescriptions*	0.0236 (0.0034)	1.02 (1.02, 1.03)	<0.0001	–	–	–
*Atypical Antipsychotic Prescriptions*	0.0193 (0.0013)	1.02 (1.02, 1.02)	<0.0001	–	–	–
*Typical Antipsychotic Prescriptions*	0.0306 (0.0027)	1.03 (1.03, 1.04)	<0.0001	–	–	–
*Obesity Diagnosis*	-0.2054 (0.0645)	0.81 (0.71, 0.92)	0.0014	–	–	–
*Lipid Disorder Diagnosis*	-0.2199 (0.0457)	0.80 (0.73, 0.88)	<0.0001	–	–	–
*Hypertension Diagnosis*	-0.1878 (0.0475)	0.83 (0.76, 0.91)	<0.0001	–	–	–
*Diabetes Diagnosis*	-0.1244 (0.0532)	0.88 (0.80, 0.98)	0.0194	–	–	–
*Sex*						
Female	–	REF	–	–	–	–
Male	0.0130 (0.0404)	1.01 (0.94, 1.10)	0.7483	–	–	–

^^^QIC reflects quasi likelihood under the independent model criterion used to assess model fit.

^+^Covariate measurement properties are described in the Methods Data section in detail.

## Discussion

In this EHR study in a large Midwestern U.S. health system of patients with schizophrenia and schizoaffective disorder with a mean follow-up time of 28 months, the rate of relapse over was 30.52%. A previous real-world database study demonstrated similar relapse rates between 30-40% within two years among patients treated with antipsychotics with serious mental illnesses^34^.

Factors which were independent predictors of relapse in this study cohort included demographic variables (age, race/ethnicity, insurance type), comorbidities (substance use disorder), prior health care resource utilization (ER visits, behavioral health inpatient visits), prior relapse, and medication-related factors (EPS and LAI prescriptions). A systematic literature review of definitions and drivers of relapse in 145 manuscripts and five guidelines identified factors which drive or reduce the rate of relapse. The most common factor in these studies associated with greater relapse risk was non-adherence, followed by stress/depression, and substance abuse. Other identified factors which were consistent with this study included treatment-related factors (e.g. side effects, dosing issues), younger age, and hospitalization/relapse history. The association between long-acting injectable use and relapse varied between studies^27^.

Overall, when comparing the demographics of study patients who experienced a relapse to those who did not, patients who experienced a relapse were *less likely* to be privately insured. The low rate of private insurance coverage is common in schizophrenia patients in the U.S. due to employment-based private insurance and the high rate of unemployment in this population. Both Medicaid- and Medicare-insured individuals had higher rates of relapse than those who were privately insured. This relationship remained even after adjustment for other factors and could potentially be due to the most severe cases ending up on publicly-funded health care, or differences in access to care related to insurance coverage. Relapse also differed by race and ethnicity, which could reflect differences in access to care, health-seeking behaviors, stigma, and/or clinical outcomes^25,35^. These demographic factors may be useful for health systems to work with payors to identify populations at risk and may have implications in the quality, continuity, and accessibility of care.

Additionally, patients who experienced relapse were less likely to have comorbid diagnoses and/or treatments for overweight/obesity/weight gain, lipid metabolism disorder, hypertension, and diabetes. These results were an interesting finding but did not remain statistically significant in the multivariable analysis. Conversely, previous studies have demonstrated a positive correlation between a number of cardiometabolic comorbidities higher costs and higher 30-day readmission rates^36^. However, the studies differed in their definitions for comorbidities, with the current study inclusive of drug treatments (i.e. statins, insulin, oral hypoglycemic agents) as a proxy for a comorbidity diagnosis, while previous studies only used diagnosis codes. Additionally, younger individuals are less likely to have diagnosed or treated comorbidities including heart disease^37^. In this analysis, younger age was associated with a higher risk of relapse, which could have been a confounder for the cardiometabolic comorbidity variable. This study also predates health system-wide initiatives to improve cardiometabolic screening among individuals with schizophrenia, so more recent data and longer follow-up may be needed to determine whether screening and detection of cardiometabolic disease leads to a change in relapse outcomes. The impact of treated vs. untreated cardiovascular comorbidities on relapse rates warrants further exploration.

Patients who experienced relapse were also more likely to have a comorbid diagnosis of substance use. The relationship between substance abuse and schizophrenia is well documented. Substance use in psychosis is associated with poorer outcomes, including increased psychotic symptoms and poorer treatment compliance. The links between substance abuse and schizophrenia are complex, with genetic vulnerability, psychosocial factors, self-medication, and medication adverse events all being potential contributors^38^.

Diagnosis and/or medication treatments for EPS were also independently associated with an increased risk of relapse. Consistent with these findings, previous research using deidentified administrative claims database data demonstrated a higher rate of all-cause and schizophrenia hospitalizations in patients with EPS^26^. Tolerability issues due to EPS from antipsychotic use have been shown to be one of the most common reasons for patient non-adherence to therapy^39,40^, which in turn is associated with the risk of relapse. Higher rates of EPS have also been shown to be related to the use of first-generation and select second-generation antipsychotics^40,41^. These agents made up a significant portion of antipsychotic use among these patients.

Further, patients who relapsed were younger, indicating more potential years, and opportunities, for progression. A previous analysis of the Nationwide Inpatient Sample data of hospitalization trends from 2005–2014 showed higher overall and increasing hospitalization rates over time for individuals 18–44 of age when compared to individuals 45–64 and 65 and over^42^. Other studies have also demonstrated a relationship between the age of onset of schizophrenia and relapse; however, we cannot assume that the younger age variable in this study reflects the younger age of onset. Patients who are younger and earlier in their disease may have lower clinical insight about their disease, which has been shown to be associated with higher rates of relapse^43,44^.

Perhaps unsurprisingly, patients who experienced relapse generally had higher rates of utilization prior to relapse, as indicated by more inpatient, emergency room and BH-specific inpatient healthcare system encounters. Patients who experienced relapse also had more prescriptions written within the system across all medication categories prior to relapse. While research has documented progressively more healthcare resource utilization in the years and months leading to a schizophrenia diagnosis among patients^25,45^, it is possible that increased utilization could similarly aid in the early detection of upcoming relapse. County programs were in place in one large county covered by the health system which identified repeat relapse patients for case management, which could have impacted the study findings.

During the preceding period, patients who experienced relapse also experienced more outcome episodes potentially signaling their future relapse, including more prior relapses, care escalations, medication switches and medication additions. Further, prescriptions for LAIs were also associated with a higher risk of relapse. This could be explained by the fact that patients who had a history of non-adherence, which was unable to be measured in this study, particularly those court-ordered for mental health treatment, and inherently were at higher risk of relapse, were primarily treated with LAIs within the healthcare system.

Overall, insurance, age, race/ethnicity, substance use diagnosis, EPS diagnosis, ER encounters, BH inpatient encounters, prior relapse, and LAI prescriptions were all considered significant predictors of relapse in the final model, when adjusting for other variables. These variables could conceivably be used to construct algorithms or models to proactively monitor patient demographic, comorbid, medication, and health care utilization parameters which place them at risk for relapse and modify their approach to care to avoid future relapse. Previous studies have demonstrated that earlier intervention in first-episode psychosis with comprehensive care can lead to improved outcomes compared to usual care^46,47^. However, identifying first-episode psychosis in a prevalence sample of health system patient patients in real-world data is problematic due to limited historical health record data. Also, the impact on hospitalization rates for relapse in these randomized controlled studies has been inconsistent. One pragmatic randomized controlled study comparing Specialized Treatment in Early Psychosis (STEP) to treatment as usual in first-episode patients within 5 years of diagnosis demonstrated significant reductions in hospitalization, mean number of hospitalizations, and mean bed days^47^. Another study titled The Recovery After Initial Schizophrenia Episode (RAISE) compared coordinated speciality care for early psychosis to usual community care and was shown to be more effective for retention in the program, improvement in their symptoms, interpersonal relationships, and quality of life^46^. They were also more involved in work and school. Future research should explore the impact of specialized or comprehensive care in a prevalent population of health system patients identified as high risk for relapse who are at mixed stages of duration of illness.

Given the high rate of relapse in patients with schizophrenia and schizoaffective disorder, additional future research should not only continue to focus on predictors of relapse but also on time to relapse after diagnosis or symptom-onset. Further, utilizing current technology that has been evidenced to offer new, innovative strategies to predict relapse is another area of future research^48,49^. Technological strategies, like smartphone digital phenotyping^48^ and mobile sensing-based deep learning models^49^, can help detect changes in behavior and symptoms in individuals with schizophrenia to ultimately prevent relapse from occurring. This technology can complement this analysis by identifying important variables to monitor and track for changes that may indicate upcoming relapse.

### Limitations

The study sample included all patients within the healthcare system with a diagnosis of schizophrenia or schizoaffective disorder in the timeframe with at least 12 months of follow-up in the system. Study sample patients had, on average, 28 months of touchpoints within AAH during the study timeframe, suggesting the study sample had more established, ongoing care within the system. While ongoing, consistent care could indicate this sample was inherently at decreased risk of relapse relative to the general population of individuals with schizophrenia or schizoaffective disorders, it is also possible that the consistent use of the system increased the likelihood of detecting relapse relative to individuals who move around for care.

It is important to note that the definition of relapse in this study reflected hospitalization (i.e. emergency department or inpatient admission encounter) for a psychiatric-related service after a 6-month baseline period of care for schizophrenia or schizoaffective disorder in the healthcare system. It is possible that, if analyzed closer, some patients categorized in this study as having experienced relapse did not truly fit this definition and vice versa. Because of the large sample size and the systematic data extraction from the healthcare EHR, it was not feasible to review each case individually, but we assume that mis-categorization, if it occurred, occurred pretty equally for both groups.

It should also be noted that much of the data used in this study was captured post-COVID-19 pandemic, an important factor that would have affected these results. Because this study included only healthcare patients who had at least 12 months of follow-up in the healthcare system, exploring COVID-19 would have provided a skewed look at the impact of the pandemic on relapse; thus we chose to explore only patient-specific predictors of relapse in this manuscript. We plan to pursue the impact of COVID-19 on relapse and other outcomes among patients with schizophrenia or schizoaffective disorder,

Some groups were smaller than others across the number of candidate covariates, particularly Other Pacific Islanders. Despite differences in the outcome in bivariate analyses, such small numbers should be interpreted with caution as findings could be misleading; however, we chose to include groups with small numbers to provide a comprehensive look at a healthcare patient sample.

Diagnosis of schizoaffective disorder versus schizophrenia at the Index Date was not included as a candidate covariate for a few reasons. First, inclusion diagnosis was only collected at one encounter (i.e. Index Date), so diagnosis could not be verified longitudinally. Also, the distinction between diagnoses of schizoaffective and schizophrenia requires behavioral health expertise for an accurate diagnosis, but inclusion diagnosis at Index Date was collected across all encounter settings, including non-behavioral health settings such as general hospital outpatient, inpatient or emergency room. Further, analysis of inclusion diagnosis at Index Date revealed three times greater likelihood of a schizoaffective diagnosis if the Index Date encounter setting was behavioral health vs. non-behavioral health. For these reasons, we felt this variable was not interpretable as a predictor.

Medication non-adherence, already mentioned as a known predictor of relapse, as defined more broadly in literature as consistent use of prescribed medication over the course of treatment, was difficult to accurately extract from medical records and thus was not included in our dataset or subsequent modeling. While EMR data offers a huge benefit to this body of literature, there are limitations to extracting information pertaining to certain types of data, like medication prescriptions. Difficulty of extraction was due not only the variety of medication prescriptions that could potentially represent adherence but also the inability to identify if patients were adherent to the different methods, dosages and timing their prescriptions required. Also, this study did not differentiate between first- and second-generation antipsychotics, despite evidenced correlations with candidate covariates, specifically EPS. Future research should explore first- and second-generation prescriptions separately, as there may be differences.

In general, the goal of this study was to identify unique predictors of relapse among a large sample of patients with schizophrenia or schizoaffective disorder. It is possible that effect modification was present among candidate covariates, but identification of these relationships was not the focus of exploration in this analysis. However, in response to significant changes to parameter estimates during model building, two interaction terms (insurance*EPS diagnosis and race/ethnicity*EPS diagnosis) were added, separately, to the final model, but neither proved to reflect effect modification. Exploration of interactions is a potential avenue for future research.

Exploring cardiometabolic-based disorders as a group could have been beneficial but we chose to look at the diagnoses separately to truly understand the risk of relapse across each individual diagnosis. We plan to explore diagnosis combinations that better represent cardiometabolic risk more comprehensively in future research.

## Conclusions

These findings reflect a large, diverse prevalence dataset to describe risk and predictors of relapse among patients with schizophrenia and/or schizoaffective disorder who had at least 12 months of post-diagnosis follow-up within the largest integrated non-profit healthcare system in the Midwest. Findings from this study contribute valuable clinical data on variable predictors of relapse and could potentially be proactively used to assess the risk of relapse for a patient with schizophrenia or schizoaffective disorder.

## Data Availability

Minimal data needed to interpret, replicate or build upon findings can be made available via request to the corresponding author.
